# Identification and Characterization of SOG1 (Suppressor of Gamma Response 1) Homologues in Plants Using Data Mining Resources and Gene Expression Profiling

**DOI:** 10.3390/genes13040667

**Published:** 2022-04-09

**Authors:** Andrea Pagano, Carla Gualtieri, Giacomo Mutti, Alessandro Raveane, Federico Sincinelli, Ornella Semino, Alma Balestrazzi, Anca Macovei

**Affiliations:** Department of Biology and Biotechnology ‘L. Spallanzani’, University of Pavia, via Ferrata 9, 27100 Pavia, Italy; andrea.pagano01@universitadipavia.it (A.P.); carla.gualtieri01@universitadipavia.it (C.G.); giacomo.mutti01@universitadipavia.it (G.M.); alessandro.raveane01@universitadipavia.it (A.R.); federico.sincinelli01@universitadipavia.it (F.S.); ornella.semino@unipv.it (O.S.); alma.balestrazzi@unipv.it (A.B.)

**Keywords:** DNA damage response (DDR), Suppressor of Gamma response 1 (SOG1), gene expression, seed development, seed germination

## Abstract

SOG1 (Suppressor of the Gamma response 1) is the master-regulator of plant DNA damage response (DDR), a highly coordinated network of DNA damage sensors, transducers, mediators, and effectors, with highly coordinated activities. SOG1 transcription factor belongs to the NAC/NAM protein family, containing the well-conserved NAC domain and five serine-glutamine (SQ) motifs, preferential targets for phosphorylation by ATM and ATR. So far, the information gathered for the SOG1 function comes from studies on the model plant *Arabidopsis thaliana*. To expand the knowledge on plant-specific DDR, it is opportune to gather information on other SOG1 orthologues. The current study identified plants where multiple SOG1 homologues are present and evaluated their functions by leveraging the information contained in publicly available transcriptomics databases. This analysis revealed the presence of multiple SOG1 sequences in thirteen plant species, and four (*Medicago truncatula*, *Glycine max*, *Kalankoe fedtschenkoi*, *Populus trichocarpa*) were selected for gene expression data mining based on database availability. Additionally, *M. truncatula* seeds and seedlings exposed to treatments known to activate DDR pathways were used to evaluate the expression profiles of *MtSOG1a* and *MtSOG1b*. The experimental workflow confirmed the data retrieved from transcriptomics datasets, suggesting that the *SOG1* homologues have redundant functions in different plant species.

## 1. Introduction

The conservation of genome integrity is a key component for the perpetuation of life. To maintain genome integrity and detect and repair DNA lesions, all living organisms activate sophisticated and interconnected mechanisms, collectively gathered under the umbrella of the DNA damage response (DDR). From an evolutionary point of view, the DDR pathway is highly conserved in eukaryotes [1,2]. The intricated DDR network is composed of DNA damage sensors, transducers, mediators, and effectors, with highly coordinated activities that can either lead to DNA repair and cell survival or trigger cell death if the damage cannot be effectively repaired [1,3]. DDR sensors are proteins able to recognize DNA damage, and this, in turn, activates a series of events (e.g., phosphorylation cascades) that lead to the regulation of downstream processes (e.g., cell cycle checkpoint, DNA repair, programmed cell death) [4]. In both animals and plants, the MRN (MRE11/RAD51/NBS1) complex is required for the recognition of strand breaks in pathways involving the main signal transducers ATM (ataxia telangiectasia mutated) and ATR (Rad3-related) kinases [1,5]. ATM and ATR are responsible for the phosphorylation of proteins, such as the histone-variant H2AX [6,7] which, in the phosphorylated form (γH2AX), acts as a DNA damage signal and recruiter of several proteins to the double-strand break (DSB) site [4,8,9]. Following damage recognition, plant cells activate DNA repair genes [8] concomitantly to blocking the progression of the cell cycle [10] to allow the damage to be repaired, or it activates the programmed cell death pathways [11] to remove the damaged cells. 

As revealed by bibliometric analysis, studies on plant DDR lag far behind mammalian research, covering only 10% of the total number of published articles on DDR [12]. This is startling because many mutations that are lethal in animals are viable in plants [13,14,15]. Although DDR is highly conserved in eukaryotes, peculiar plant-specific features are also present [1,2,3]. For instance, SOG1 (Suppressor of gamma response 1) is considered to be the functional homolog of the mammalian p53 (the main DDR effector), and hence, the master-regulator of plant DDR [16,17,18]. SOG1 is a transcription factor (TF) belonging to the NAC (originally characterized from consensus sequences from petunia NAM and *Arabidopsis* ATAF1, ATAF2, and CUC2) family. In the model plant *Arabidopsis thaliana*, more than 100 genes belonging to this family of TFs have been identified and divided into ten major groups, making this protein family one of the largest in plants [19,20,21]. Although it was demonstrated that NAC TFs play critical roles in different processes such as environmental stress responses, xylem cell specification, lateral root formation, or the establishment of the shoot apical meristem, the function of many NAC proteins is still uncertain [19,22,23]. The SOG1 function was first identified as a *sog1* allele able to suppress the growth arrest induced by γ-ray in the *A. thaliana* UV-hypersensitive (*uvh*)1 mutant [16,24]. Aside from the well-conserved NAC domain, the C-terminus of AtSOG1 is characterized by the presence of five serine-glutamine (SQ) motifs, which are preferential targets for phosphorylation by ATM and ATR [9,25]. The DNA damage-dependent SOG1 hyperphosphorylation detected in wild-type plants disappeared in transgenic plants bearing a mutated SOG1 showing serine-to-alanine substitutions at all five SQ motifs, suggesting that one or more of the SQ motifs are effective targets for the hyperphosphorylation [25]. Considering that these motifs are conserved in eudicots, monocots, an ancient flowering plant (*Amborella trichopoda*), and gymnosperms, Yoshiyama et al. [17] proposed that SOG1 had already been acquired starting from gymnosperms. However, more recent studies also reported the presence of SOG1 orthologues in lower plants such as *Physcomitrella patens* [26], thus placing its origin in nonvascular plants [27]. SOG1 is the first TF whose function was associated with DDR in plants. Nonetheless, more recently, other TFs from the NAC family (e.g., NAC044, NAC085) have been associated with DDR functions and hypothesized to be involved in the control of mitotic genes [28,29]. Among these, NAC044 (a DDR mediator) is considered to be a close homolog of SOG1, as nac044 mutants showed reduced sensitivity to treatments that induce DSBs [29]. When DSBs occur, SOG1 is activated through ATM-mediated phosphorylation, similarly to animal p53. As a master regulator, it drives the cell fate towards cell cycle arrest, DNA repair, apoptosis or senescence, and endoreduplication. Despite its similar function to the mammal p53, these transcription factors lack significant amino acid sequence similarity and are examples of divergent proteins. Similar to mammals where p53-independent pathways are present [30], SOG1-independent pathways were also reported in plants [28]. These include the E2Fs–RBR1 (RETINOBLASTOMA-RELATED1) complex, which can interact with DREAM complexes (considered as master regulators of the cell cycle) to repress gene expression in G1 [3,31]. On the other hand, RBR1 interacts also with NAC044, and the disruption of this interaction can impair cell death [32].

So far, most studies on the SOG1 function in plants were conducted in *Arabidopsis* [1,16,25,28,33,34,35,36]. To expand the knowledge on plant-specific DDR, it is necessary to investigate other SOG1 orthologues, considering that other plants can also possess multiple homologues [27]. Moreover, SOG1 functions may differ in different species, also considering that in *Arabidopsis,* distinct sets of target genes can be differentially regulated by SOG1 in different cell types [17,37]. Hence, in this study, we aimed to identify plants where multiple SOG1 homologues are present, along with evaluating the function of these genes, taking advantage of RNA-seq and microarray databases. In addition to in silico data mining, an experimental system was specifically set using *Medicago truncatula* seeds and seedlings exposed to stimulating or damaging treatments, and the expression profiles of the two *SOG1* genes (*MtSOG1a*, *MtSOG1b*) were evaluated. 

## 2. Materials and Methods

### 2.1. Phylogenetic Analysis 

A total of 72 SOG1 putative orthologue sequences from 49 species were retrieved from Phytozome (vs. 12.1, http://www.phytozome.net/, accessed on 19 March 2022) using BLASTp (with default parameters) starting from the A. thaliana SOG1 peptide sequence (AT1G25580). The sequences with an expected value lower than 100 were kept and aligned with MAFFT (Multiple Alignment Fast Fourier) using the FFT-NS-i algorithm [38]. Only sequences containing the NAC domain and the SQ motives were retained, resulting in a total of 68 sequences from 47 species. 

The phylogenetic analysis was computed with IQ-Tree with 1000 Ultrafast bootstrap [39] using the JTT model as the substitution model [40] with invariant sites (+I) and *gamma* distributed rates with four categories (+G4) as found by ModelFinder [41]. The tree was rooted with midpoint rooting. The NAC domain was annotated through the HMMER web server [42] over the Pfam database with default parameters and the SQ motifs were searched with a regex search. Tree annotation and visualization were performed using the ETE3 python package [43].

### 2.2. Gene Expression Data Mining

Data for gene expression profiles were retrieved from the BAR Toronto eFP browser (http://bar.utoronto.ca/, accessed on 19 March 2022, [44]), a visual analytic tool used to explore multiple levels of expression data in plants. Specifically, data were retrieved for the following species: *M. truncatula* [45], *Glycine max* [46], *Kalanchoë fedtschenkoi* [47], and *Populus trichocarpa* [48]. This choice was based on data availability for species containing multiple SOG1 homologues and represented in the BAR Toronto eFP browser. The “absolute” mode was used, indicating the expression levels directly associated with the most intense signal recorded for each gene. The intensities obtained through eFP (electronic fluorescent pictography) were normalized using the MAS5 algorithm, which isolates individual signals by removing the background noise [49]. For all species, the data are presented as FPKM (fragments per kilobase of exon model per million reads mapped)-normalized. For *M. truncatula*, additional data on gene expression were collected from the Phytozome Gene Atlas [50] to cover aspects related to gene expression during symbiotic conditions. In all the studies, samples were analyzed in triplicate while only the mean values are available in this database, and hence were retrieved and used to represent the data as heatmap models generated using Shinyheatmap (http://shinyheatmap.com/, accessed on 19 March 2022, [51]). The expression values are represented as Z-scores, a numerical measure that describes the relationship of a value to the mean of a group of values. The following parameters were set for all the generated heatmaps: Blue for low values, white for mid-values, red for high values, no clustering, no trace, a Euclidean distance metric, a complete linkage algorithm, and row scaling. 

### 2.3. In Silico Analyses of SOG1 Homologues in M. truncatula

The genomic, transcript, coding, and peptide sequences of *M. truncatula* SOG1 homologues were retrieved from Phytozome. The PhytoMine (https://phytozome.jgi.doe.gov/phytomine/begin.do, accessed on 19 March 2022) tool was used to find the precise positions of the exons on genomic sequences, average length, and distribution. Pfam (http://www.pfam.xfam.org, accessed on 19 March 2022) and InterPro (http://www.ebi.ac.uk/interpro/, accessed on 19 March 2022) were used to confirm and locate the NAM/NAC characteristic domain. Alignment of the MtSOG1a (Phytozome accession No. Medtr5g053430) and MtSOG1b (Phytozome accession No. Medtr1g093680) was performed using CLUSTALW (https://www.ebi.ac.uk/Tools/msa/clustalo/, accessed on 19 March 2022). 

The STRING (https://string-db.org/, accessed on 19 March 2022) online database (vs.11.0) was used to identify the AtSOG1, MtSOG1, GmSOG1, and the PtSOG1 putative protein–protein interactors. The polypeptide sequence was used as a query and the default STRING settings were applied, displaying all the predicted interactors with a score higher than 0.4 with confidence-based links and including results from all the available evidence sources. Accession numbers were retrieved from the list of interactors (STRING codes) displayed as the output of the STRING query (one per species since the different SOG1 genes in the same species have almost perfectly overlapping interactomes). Starting from the list of interactors in *A. thaliana*, homologues in *M. truncatula*, *G. max*, and *P. trichocarpa* have been retrieved on Phytozome BioMart. 

Promoter analysis was carried out for the *MtSOG1a* and *MtSOG1b* genes to identify putative *cis*-acting regulatory elements. The sequences of the promoter regions were retrieved from NCBI Gene (https://www.ncbi.nlm.nih.gov/gene/?term=, accessed on 19 March 2022), displaying the region within 1000 bp upstream of the transcription start site. Using the retrieved sequences as queries, the putative cis-acting regulatory elements were identified and described using the New PLACE database (https://www.dna.affrc.go.jp/PLACE/?action=newplace, accessed on 19 March 2022).

### 2.4. Plant Materials and Treatments 

*M. truncatula* commercial seeds (Jemalong cultivar) were provided by Fertiprado L.d.a., Vaiamonte (Monforte, Portugal). For imbibition, seeds were placed in Petri dishes (30 seeds per dish) on a layer of filter paper moistened with 2.5 mL H_2_O and imbibed for 24 h. Samples were collected at 0, 2, 4, 6, 8, 12, 16, and 24 h of imbibition. Dry-back treatments were carried out on two subpopulations of 24h-imbibed seeds: (i) Primed seeds (P) not displaying radicle protrusion and (ii) over-primed seeds (OP) displaying radicle protrusion (2 mm in length). A total of 30 seeds were distributed into Petri dishes and covered with a layer of absorbing paper and a layer (~20 g) of silica beads (Disidry^®^ Orange Silica Gel, The Aerodyne, Florence, Italy). Petri dishes were sealed with parafilm. P and OP samples were collected after 0, 2, 4, and 6 h of dehydration. Sample collection during imbibition and dry-back was carried out by excising the embryo axis along with the radicle protrusion, when present.

The relative water content (RWC) was calculated during imbibition (0, 2, 4, 6, 8, 12, 16, and 24 h) and dry-back of P and OP seeds (0, 2, 4, and 6 h), according to the formula RWC [%] = [(Fw − Dw)/Fw] × 100. For each sample (3 replicates of 20 seeds each), fresh weight (Fw) was measured at the indicated timepoints. Dry weight (Dw) was measured after overnight dehydration at 80 °C [52].

Additional treatments were carried out on *M. truncatula* seeds using 25 μM camptothecin (CPT, Sigma-Aldrich, Milan, Italy) and 25 μM NSC120686 (NSC, 2-chloro-6-fluorobenzaldehyde 9H-fluoren-9-ylidenehydrazone) provided by the National Cancer Institute (Bethesda, United States), following the experimental design reported by Gualtieri et al. [53]. Because these compounds are dissolved in dimethyl sulfoxide (DMSO) (Sigma-Aldrich, Milan, Italy), specific controls, corresponding to each concentration (0.17%, 0.23%, and 0.29%) used in the indicated treatments, were set up. The treatments were applied to seeds placed in Petri dishes (30 seeds per dish) containing a filter of blotting paper moistened with 2.5 mL H_2_O (non-treated control) or indicated solutions (CPT, NSC, CPT+NSC, DMSO controls). Seedlings were harvested after 7 days and frozen in liquid nitrogen.

All Petri dishes (for seed imbibition and CPT/NSC treatments) were kept in a growth chamber at 22 °C under light conditions with a photon flux density of 150 μmol m^−2^ s^−1^, a photoperiod of 16/8 h, and 70–80% relative humidity.

### 2.5. Quantitative RealTime-PCR Profiling 

Total RNA was isolated from *M. truncatula* seeds and seedlings as previously described [53,54]. Samples were ground in liquid nitrogen, mixed with 550 μL Extraction Buffer (0.4 M LiCl, 0.2 M Tris pH 8.0, 25 mM EDTA, 1% SDS), and 550 μL chloroform. Samples were centrifuged at 10,000 rpm for 3 min at 4 °C, followed by the addition of phenol-chloroform to the supernatant and centrifuged again. LiCl (1/3 volume, 8 M) was added to the supernatant, incubated at 4 °C for 1 h, and centrifuged. The pellet was washed with 70% ethanol, air-dried, and suspended in DEPC (diethylpyrocarbonate) water. A DNase (ThermoFisher Scientific, Monza, Italy) was performed, as indicated by the manufacturer. RNA was quantified using NanoDrop (Biowave DNA, WPA, ThermoFisher Scientific). Subsequently, cDNAs were obtained using the RevertAid First Strand cDNA Synthesis Kit (ThermoFisher Scientific) according to the manufacturer’s suggestions. 

Quantitative RealTime-PCR reactions were performed with the Maxima SYBR Green qPCR Master Mix (ThermoFisher Scientific) according to the supplier’s indications, using a Rotor-Gene 6000 PCR apparatus (Corbett Robotics Pty Ltd., Brisbane, Queensland Australia). Amplification conditions were as follows: Denaturation at 95 °C for 10 min, and 45 cycles of 95 °C for 15 s and 60 °C for 60 s. Oligonucleotide sequences (MtSOG1a-fw: TGGTGCGAAGGGACAGATAA, MtSOG1a-rev: TCACACCGGACAATGCGTC, MtSOG1b-fw: GGAAGCCGAAAGCGTAGAAA, MtSOG1b-rev: TTCTGAAGCCCFTTCAAGAG) were designed using Primer3Plus1 (https://primer3plus.com/, accessed on 19 March 2022) and verified with Oligo Analyzer.2 (https://eu.idtdna.com/pages/tools/oligoanalyzer, accessed on 19 March 2022). Relative quantification was carried out using actin-related protein 4A (Act, Phytozome accession No. Medtr3g095530; FW: TCAATGTGCCTGCCATGTATG, REV: ACTCACACCGTCACCAGAATC) and elongation factor 1α (ELF1α, Phytozome accession No. Medtr6g021805; FW: GACAAGCGTGTGATCGAG, REV: TTTCACGCTCAGCCTTAA) as reference genes [53]. Raw fluorescence data were used to estimate PCR efficiency (E) and threshold cycle number (Ct) for each transcript quantification, while the Pfaffl method [55] was applied to calculate the transcript’s relative quantification. All reactions were performed in triplicate. For CPT/NSC data, the results are also presented as the fold change (FC), where values for each treatment were normalized to the corresponding DMSO control.

### 2.6. Statistical Analyses 

Data were subjected to an Analysis of Variance (ANOVA), and the statistical significance of mean differences was determined. For the CPT/NSC stress treatments, Student’s *t*-test was applied (*, *p* < 0.05) to compare the treatments with the non-treated control. For the seed imbibition experiments, data were analyzed by a two-way ANOVA and the Tukey-Kramer test, using the R package ‘Shiny’, a web-based program freely available online (https://houssein-assaad.shinyapps.io/TwoWayANOVA/, accessed on 19 March 2022), developed by Assaad et al. [56]. 

Correlation analysis was carried out using Microsoft Excel Spreadsheet Software | Microsoft 365 to generate scatter plots. The correlation (r) measures the amount of linear association between two variables, where r is always between −1 and 1 inclusive. The R2 values were interpreted as follows: Negligible correlation (0–0.1), weak correlation (0.1–0.39), moderate correlation (0.4–0.69), strong correlation (0.7–0.89), and very strong correlation (0.9–1.0) [57]. 

## 3. Results and Discussion 

### 3.1. Distribution of Multiple SOG1 Homologues within the Plant Kingdom 

**Figure 1:**
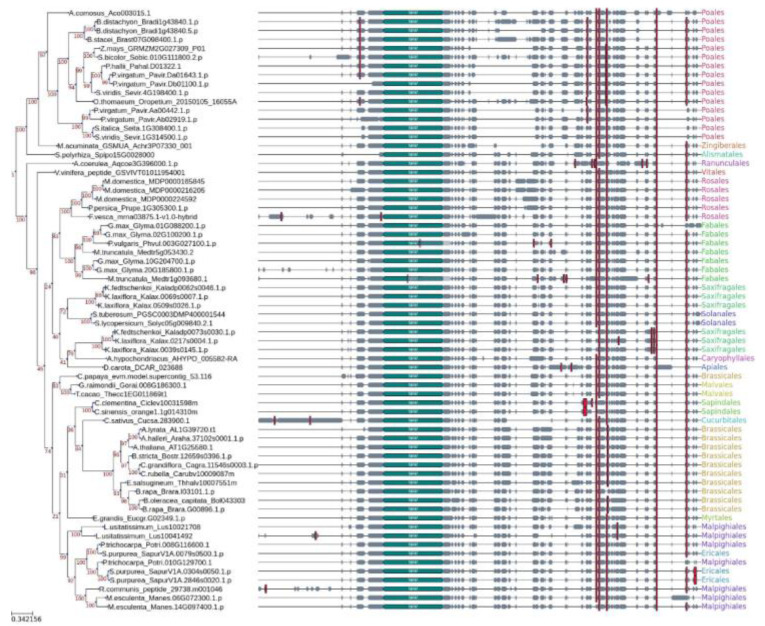
Phylogenetic Maximum Likelihood tree of the SOG1 gene family with the corresponding multiple sequence alignment. Bootstrap values are shown in red. The NAM domain is annotated in light blue and the SQ motifs are highlighted in red. The Order of each species is written in color-coded text on the right.

Phylogenetic analysis was performed using the 68 SOG1 homologous sequences retrieved as explained in the material and methods section (Figure 1). The NAM (NAC) domain was found in all species of the analyzed dataset and the phylogenetic tree revealed two main groups in which Monocots and Eudicots species fall, respectively. 

The first group contains mainly Poales that are characterized by an overall high number of SQ motives. All the relationships in the Monocots species are resolved and agree with the known species’ phylogeny as Alismatales is the sister group to the Zingiberales and Poales clades [58]. Interestingly, a duplication event may have occurred in the ancestor of Poaceae (the node comprising *Zea mays*, *Panicum hallii*, *P. virgatum*, *Sorghum bicolor,* and *Oropetium thomaeum*) and may have been lost in non-Panicoideae lineages (*Brachypodium distachyon* and *B. stacei*). Indeed, all these orthologues miss the last SQ motif otherwise perfectly conserved in other Poaceae sequences. This may be in line with previous reports where direct comparison of Poaceae paralogous gene pairs simultaneously duplicated indicates great variation in their evolutionary rates among whole genomes [59]. 

Differently, the Eudicots topology is not clearly resolved. Specifically, relationships within the same family are generally clear, whereas relationships across different families show low bootstrap support and sometimes do not overlap with the species’ phylogeny. There are lineage- and species-specific duplications events characterizing, for example, Brassicales species or the apple. Four of the SQ motifs are almost perfectly conserved in all sequences, being absent only in specific clades such as *Aquilegia coerula* and *Citrus* species or in sequences arising from a duplication event such as the Saxifragales, *Brassica rapa,* and *Manihot esculenta* sequences, perhaps suggesting subfunctionalization of the homologue genes.

The phylogenetic structure among the Fabids in our dataset is well resolved, with the Fabales species being a sister group of the Rosales. In particular, the two homologue sequences in *M. truncatula* (MtSOG1a and MtSOG1b) are placed as an outgroup in both the sub-clades of the Fabales groups, which are mainly represented by paralog sequences of soybeans (*G. max*). The placement of *M. truncatula* (MtSOG1a, Accession no. Medtr5g053430) is in line with known species’ phylogenies, being an outgroup of both Phaseoleae species (*G. max* and *Phaseolus vulgaris*). On the other hand, the second gene (MtSOG1b, Accession no. Medtr1g093680) presented five more SQ domain duplications than the one found in the majority of the species in our dataset and one of them was found within the NAM domain.

For the majority of the analyzed species, it is possible to observe the presence of only a single SOG1, except for *B. rapa*, *G. max*, *K. fedtschenkoi*, *K. laxiflora*, *Linum usitatissimum*, *Malus domestica*, *M. esculenta*, *M. truncatula*, *P. thricocarpa*, *P. virgatum*, *Setaria italica*, *S. viridis*, and *Salix purpurea*, which presented multiple homologues. Among these, *G. max*, *K. laxiflora*, and *P. virgatum* are the species with the highest number of putative SOG1 sequences (four genes). Other reports have identified SOG1-like proteins in different plants belonging to eudicot, monocot, and gymnosperms [17,18], while a more recent report identified two SOG1 orthologues in the moss *P. patens*, thus placing the origin of this gene family in nonvascular plants [27]. 

### 3.2. Expression Profiles of SOG1 Genes Retrieved from Transcriptomics Repositories

Considering that we identified several species presenting multiple SOG1 genes, we then wanted to investigate the expression of these genes in different backgrounds. For this purpose, we leveraged the transcriptomics datasets deposited in the Bio-Analytic Resource for Plant Biology (BAR). Among the different species with multiple SOG1 homologues, *M. truncatula*, *G. max*, *K. fedtschenkoi*, and *P. trichocarpa* are the only ones available in BAR. Hence, gene expression data were mined for these species, spanning different tissues and growth conditions (relative to the specific references to studies in which the data were generated), and heatmaps were built to compare the expression of the different homologues. The gene nomenclature was decided based on sequence similarity with the AtSOG1 gene (Figure S1, Table S1). 

**Figure 2:**
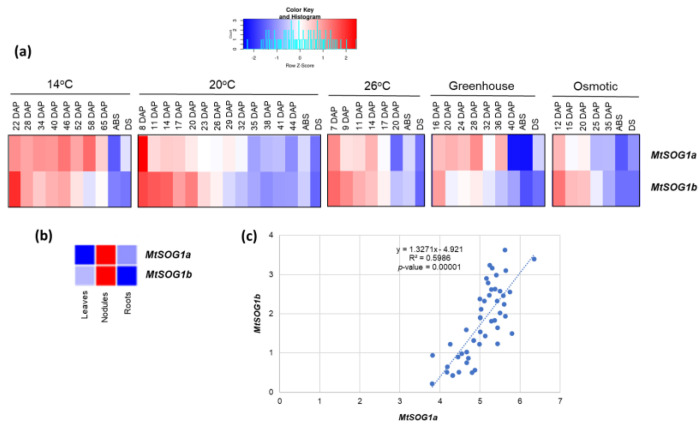
(**a**) Expression profiles of the *MtSOG1a* (Medtr5g053430) and *MtSOG1b* (Medtr1g093680) genes in *M. truncatula*. Data were collected from http://bar.utoronto.ca/, accessed on 19 March 2022 and heatmaps were generated using the Shinyheatmap (http://shinyheatmap.com, accessed on 19 March 2022). (**b**) Expression profiles in roots, leaves, and nodules of 4-week-old plants grown under greenhouse conditions. (**c**) Correlation analysis of *MtSOG1a* and *MtSOG1b* gene expression values.

For *M. truncatula*, data on the effect of stress during seed maturation and pod abscission (ABS) as well as data on dry seeds (DS) were recovered from Righetti et al. [45]. The following treatments were taken into consideration: Standard conditions (20/18 °C), low temperature (14/11 °C), high temperature (26/24 °C), osmotic stress (20/18 °C; −0.1 MPa), and greenhouse conditions (variable temperature and light). The generated heatmap (Figure 2a) shows that both *MtSOG1a* (Medtr5g053430) and *MtSOG1b* (Medtr1g093680) genes are generally highly expressed during the early seed developmental stages, independent of the temperature conditions. Similarly, the expression of both genes decreases while approaching seed maturation, with the lowest values registered in DS and ABS. The same expression pattern is also maintained during osmotic stress and greenhouse experiments (including the expression of genes in leaves, nodules, and roots of four sufficiently old plants, Figure 2b), in line with the moderately positive (R^2^ = 0.59) correlation observed (Figure 2c). An interesting fact is related to the high expression of the two genes in nodules, suggesting enhanced activity under symbiosis. However, under different circumstances, a study on *A. thaliana* evidenced that SOG1 regulates the crosstalk between DDR and the immune response [60], so it may be that this crosstalk could also be activated during symbiotic relations. During seed maturation, water is progressively lost to prepare the seed to become desiccation-tolerant, a characteristic that allows prolonged survival in the dry state until conditions become optimal for germination. This process is accompanied by extensive transcriptomic changes and is highly regulated through the activity of different phytohormones [61,62]. Specifically, an optimal balance between cell-cycle regulation and DNA repair is necessary to limit, as much as possible, the accumulation of DNA damage, known to occur during the desiccation phase [63,64]. A transcriptome profiling study carried out on *M. truncatula* seeds during the transition from desiccation-sensitive to desiccation-tolerant stages evidenced massive repression of genes belonging to cell cycle and DNA processing, indicating a general downregulation of metabolic processes and cellular activity at late seed maturation stages [61]. This is in line with a more recent study where E2Fs (transcription factors acting as regulators of cell proliferation) were shown to be involved in cell differentiation during seed and embryo development by restricting the seed maturation program until the cell proliferation phase is completed [65]. Hence, the pattern of expression of *MtSOG1a/b* is in agreement with a more active DDR during the initial phases of seed maturation while, when the water content is diminished at later phases, DDR is less required, and will subsequently be reactivated during seed imbibition and germination. 

**Figure 3:**
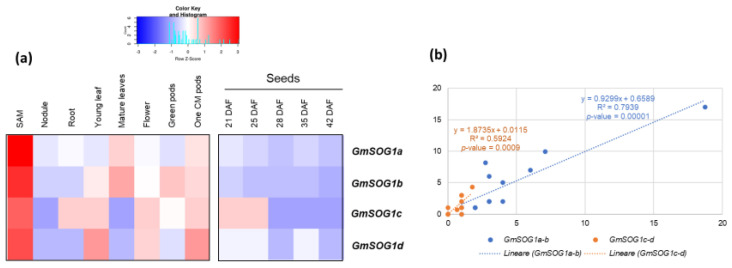
(**a**) Expression profiles of the *GmSOG1a* (Glyma.02G100200), *GmSOG1b* (Glyma.20G185800), *GmSOG1c* (Glyma.10G204700), and *GmSOG1d* (Glyma.01G088200) genes in *G. max*. Data were collected from http://bar.utoronto.ca/, accessed on 19 March 2022 and heatmaps were generated using the Shinyheatmap (http://shinyheatmap.com, accessed on 19 March 2022). (**b**) Correlation analysis between *GmSOG1a*, *GmSOG1b*, *GmSOG1c*, and *GmSOG1d* gene expression values.

In the case of soybean, four *GmSOG1* homologues were identified: *GmSOG1a* (Glyma.02G100200), *GmSOG1b* (Glyma.20G185800), *GmSOG1c* (Glyma.10G204700), and *GmSOG1d* (Glyma.01G088200). Data available on different plant tissues (roots, nodules, leaves, flowers, pods, SAM) and seed maturation stages (given as days after flowering, DAF) were retrieved from the *G. max* RNA-seq atlas [46]. The gene expression data-mining approach showed that all four genes were highly expressed in the shoot apical meristems (SAM), whereas in seeds, the expression patterns were similar to what was observed in *M. truncatula*, with all genes having a decreasing expression with an increase in stages of seed maturation (Figure 3a). Furthermore, in this case, moderate (R^2^ = 0.59) and strong (R^2^ = 0.79) positive correlations between each two gene copies were evidenced (Figure 3b), indicating co-expression of the homologues in different tissues. The same expression pattern of *GmSOG1*s and *MtSOG1*s during seed development ascertain, once more, the implication of DDR in this process. As for the observed upregulation of *GmSOG1*s in SAM, this is in agreement with studies from *Arabidopsis* indicating that the high *SOG1* expression detected in meristematic cells is associated with immediate transcriptomic changes occurring in response to DNA damage [33,34]. 

**Figure 4:**
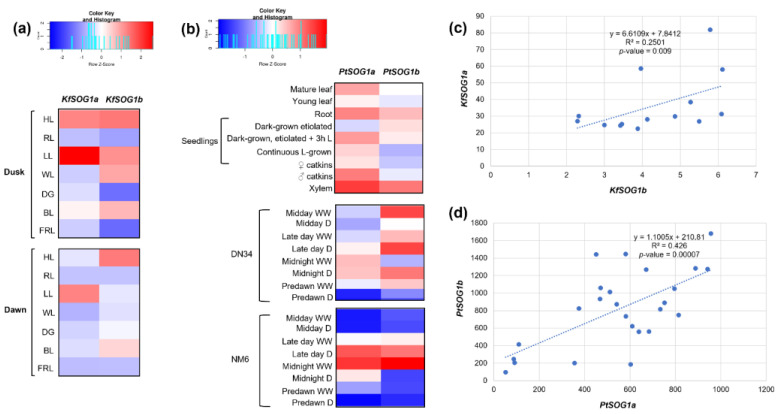
(**a**) Expression profiles of the *KfSOG1a* (Kaladp0062s0046) and *KfSOG1b* (Kaladp0073s0030) genes in *K. fedtschenkoi*. (**b**) Expression profiles of the *PtSOG1a* (Potri.008G116600) and *PtSOG1b* (Potri.010G129700) genes in *P. trichocarpa*. Data were collected from http://bar.utoronto.ca/, accessed on 19 March 2022 and heatmaps were generated using the Shinyheatmap (http://shinyheatmap.com, accessed on 19 March 2022). (**c**) Correlation analysis between *KfSOG1a* and *KfSOG1b* gene expression values. (**d**) Correlation analysis between *PtSOG1a* and *PtSOG1b* gene expression values.

Data from *K. fedtschenkoi* were retrieved from a light-responsive expression atlas, where light quality and intensity were evaluated [47]. Plants were grown under different conditions: Control (WL), blue light (BL), red light (RL), far-red light (FRL), dark-grown (DG), low light (LL), and high light (HL), and samples were collected at two-time points, dawn (2 h before light period) and dusk (2 h before dark period). The generated heatmap (Figure 4a) indicates that, at dusk, both *KfSOG1a* (Kaladp0062s0046) and *KfSOG1b* (Kaladp0073s0030) were highly expressed in LL and HL while low expressions were registered for RL, DG, and FRL. Different profiles are observed at dawn when HL induced higher expression of *KfSOG1b* while *KfSOG1a* was more expressed at LL. These slight differences also impacted the correlation analysis, where only a weak (R^2^ = 0.25) positive correlation was observed between the expression profiles of the two homologues (Figure 4c). It is well-known that the photosynthetic apparatus generates reactive oxygen species (ROS), especially when plants are exposed to excessive light [66,67]. In turn, ROS may activate DDR players to protect the cells from oxidative damage [3]. For instance, the SIAMESE-RELATED cyclin-dependent kinase inhibitors SMR5 and SMR7 were shown to be specifically activated in an ROS-dependent manner after high-light treatment [68]. Hence, the changes in the *KfSOG1a/b* gene expression hereby observed in response to light treatment may follow a similar pathway of activation by ROS. 

As for poplar (*P. trichocarpa*), data from greenhouse-grown plants, in terms of different tissues and drought treatments, were made public by Wilkins et al. [48]. For the drought experiments, plantlets were grown under 16 h light/8 h dark conditions and fully expanded leaves were sampled at midday, late day, midnight, and predawn, from stressed (D) and control (WW) clones of DN34 and NM6 poplar saplings. When considering plant tissues, both *PtSOG1a* (Potri.008G116600) and *PtSOG1b* (Potri.010G129700) are highly expressed only in xylem and roots, while *PtSOG1a* is more expressed than *PtSOG1b* in male catkins and mature leaves (Figure 4b). A very similar expression of the two genes is observed during the drought treatments, specifically for the NM6 clones, where both genes are highly expressed at late day D and midnight WW timepoints while they are downregulated in the remaining samples. The correlation analysis indicates a moderate positive correlation (R^2^ = 0.42) between the expression patterns of the two genes (Figure 4d). Among DDR downstream pathways, cell cycle regulation is a key player to modulate plant growth and development when plants have to cope with multiple stresses, including drought. This specific type of stress can be accompanied by the downregulation of the cell cycle regulators [69,70]. Moreover, specific studies with DDR mutants, such as the Arabidopsis *uvh6-1*, showed that the homologous recombination frequency is impaired in mutants mildly affected by drought [71]. 

Overall, the correlation analyses of the transcriptomic data retrieved for *M. truncatula*, *G. max*, *P. trichocarpa*, and *K. fedtschenkoi* indicate that the *SOG1* homologues identified in a given species tend to have similar expression patterns. Based on the data gathered from RNA-seq repositories, among the various conditions and physiological processes in which the genes are highly active, we can cite early seed development, highly proliferative tissues (e.g., SAM), light, and drought stresses.

### 3.3. In Silico Characterization of M. truncatula MtSOG1a and MtSOG1b Homologues

**Figure 5:**
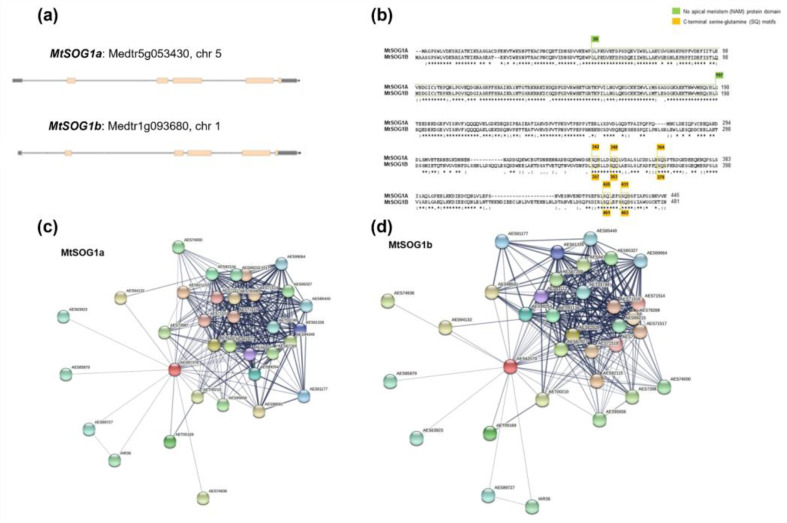
(**a**) Schematic representation of *MtSOG1a* and *MtSOG1b* gene organization as evidenced in the Phytozome (https://phytozome.jgi.doe.gov/, accessed on 19 March 2022) genome browser. Exons are presented as orange boxes. (**b**) Alignment of MtSOG1a and MtSOG1b protein sequences performed using ClustalW. The presence of NAM (No Apical Meristem) and SQ (Serine, Glutamine) motifs is evidenced in green and orange boxes, respectively. Putative protein–protein interaction networks generated by STRING for (**c**) MtSOG1a and (**d**) MtSOG1b proteins.

To further consider the SOG1 homologues, a more detailed investigation was carried out in the model legume *M. truncatula*, namely MtSOG1a (Medtr5g053430, 60.1% similarity to AtSOG1) and MtSOG1b (Medtr1g093680, 53.46% similarity to AtSOG1) (Table S1). From a genomic point of view, *MtSOG1a* is localized on chromosome 5 while *MtSOG1b* is on chromosome 1. The *MtSOG1a* genomic sequence is 4743 bp long while the length of its transcript, coding, and peptide sequence is 1744 bp, 1329 bp, and 442 aa, respectively. Concerning *MtSOG1b*, its genomic sequence is 5664 bp long whereas the length of its transcript sequence is 1889 bp, and its coding and peptide sequences are 1446 and 481 aa long. Figure 5a shows the transcript organization and chromosome location of the two *MtSOG1* genes. It is thus possible to observe that both genes have five functional exons (orange boxes). Moreover, to integrate the analysis of the expression profiles retrieved from BAR, an analysis of the *MtSOG1a* and *MtSOG1b* promoter regions was carried out to identify putative *cis*-acting regulatory elements. This analysis identified a total number of 265 and 259 putative *cis*-acting regulatory elements, respectively (Table S2). Among them, 69 and 64 types of elements were identified, respectively, with 43 (65%) of them identified in the promoter regions of both genes. These included the most recurrent ones (with more than 10 occurrences in either promoter), such as CAAT promoter consensus sequences (CAATBOX1), CACT tetranucleotides (CACTFTPPCA1), GATA boxes (GATABOX), Dof protein core binding sites (DOFCOREZM), and ARR1-binding elements (ARR1AT). The most represented putative *cis*-acting elements are associated with light responses in shoot and leaves (GATABOX, GT1CONSENSUS, IBOXCORE, etc.) and seed metabolism (DOFCOREZM, EBOXBNNAPA, MYCCONSENSUSAT, etc.) (Table S2). Given the relevance of *cis*-acting elements for the regulation of gene expression, this level of analysis needs to be considered to contextualize possible differences in the tissue- and condition-related expression patterns of the *SOG1* homologues. Consistently with the overall results obtained by transcriptome data mining, various putative *cis*-acting elements in the promoters of both genes are reported to be associated with the shoot and seed tissues and with response to light and drought stress.

A further level of analysis was dedicated to investigating the protein organization and putative interactors. The alignment between MtSOG1a and MtSOG1b amino acid sequences shows that the two sequences have a high percentage of similarity (69.7%). A schematic representation of the alignment between the two sequences is shown in Figure 5b, along with evidencing the presence of conserved protein domains. The NAM domain and the serine-glutamine (SQ) motifs on the C-terminal region are shown in green and orange boxes, respectively. In the MtSOG1A (445 aa) and MtSOG1B (481 aa) sequences, the NAM domain is located starting from aa 59 to aa 197, whereas the SQ motifs are located at positions 342, 348, 364, 425, and 431 at the C-terminal region of MtSOG1a. 

An extensive in silico protein interactome analysis was then carried out using STRING, a database that collects, scores, and integrates publicly available sources of information regarding protein–protein interactions, complemented with bioinformatics predictions [72]. This analysis was first focused on *M. truncatula*, compared to Arabidopsis, but was subsequently also extended to other species for which data were available (soybean, poplar). The interactomes of MtSOG1a (Figure 5c) and MtSOG1b (Figure 5d) are identical, evidencing 32 putative protein interactors with scores between 0.7 and 0.4 (Table S3). Most of the predicted interactors are involved in DDR, e.g., WEE1 (AES82115), ATR (AES62927), ATM (AES71516), RAD51 (AES92134), ERCC1 (AES61326), UVH1 (AES94349), KU80 (AES87269), and NBS1 (AES99064), to cite just a few. When comparing the MtSOG1a/b with the AtSOG1 interactome, it was possible to observe that many of the predicted interactors are also common to Arabidopsis; although, for the latter, the list is substantially richer mostly due to the more abundant data available for *A. thaliana*. Concerning soybean SOG1 homologues, STRING predicted 82 putative interactors for GmSOG1a, 83 for GmSOG1d, and 85 for GmSOG1b and GmSOG1c. In addition, in the case of soybean, the interactomes of the SOG1 homologues were largely overlapping in terms of interactors and scores. Similar numbers were also predicted in the case of poplar (81 and 83 putative interactors for PtSOG1a and PtSOG1b, respectively, with largely overlapping lists). When comparing the predicted interactors of all four analyzed species, we were able to pinpoint some accessions common to all species (e.g., ATM, ATR, WEE1, DER1, RAD51, SMG7, ERCC1, GTF2H2, RAD4, DDB2, UVH1, KU70, KU80, SUV2, RAD17, POLH, NBS1) while others (e.g., CDC45, REV1, CYCA3;3, E2F1, NAC103, NAC065, found only in Arabidopsis) turned out to be species-specific (Table S3). The obtained results are in agreement with extensive transcriptomic data gathered using a DREM (Dynamic Regulatory Events Miner) model reporting that SOG1 is the major activator of DDR, directly targeting the most strongly up-regulated genes, including transcription factors, DNA repair factors, and early cell cycle regulators [28]. 

Overall, the retrieved data indicate that many of the predicted SOG1 interactors can be found across species, and a large majority have ascertained functions in DDR and DDR-downstream pathways (e.g., DNA repair, cell-cycle checkpoint). It is also important to note that the interactomes of different SOG1 genes in the same species are largely overlapping and to underline that many high-score interactors recur in all the retrieved interactomes, indicating the reliability of STRING analysis for literature data mining.

### 3.4. MtSOG1a and MtSOG1b Genes Are Upregulated during Seed Imbibition, Priming, and Genotoxic Stress Treatments

To integrate the data mining approach with further experimental results, we investigated the expression levels of *MtSOG1a* and *MtSOG1b* in peculiar systems, such as seed priming/over-priming [73,74] and specific genotoxic stress imposed by treatments with camptothecin (CPT, topoisomerase I inhibitor) and NSC120686 (tyrosyl-DNA phosphodiesterase 1 inhibitor) [53]. Both systems have been previously proven to induce different types of DNA damage and activation of DDR pathways [53,73,74]. 

**Figure 6:**
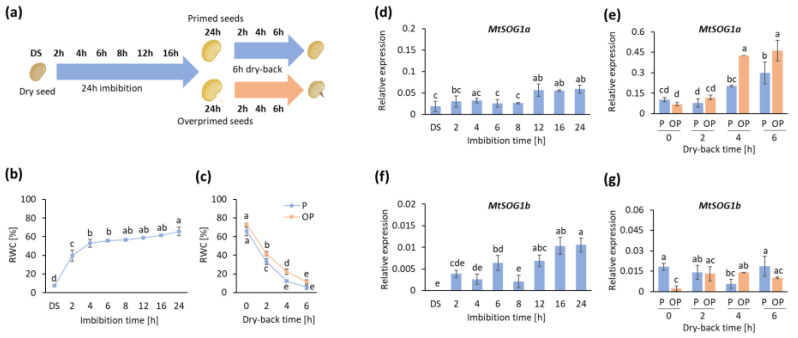
(**a**) Experimental design for the seed priming/over-priming system. Dry seeds and imbibed seeds were collected at 2 h, 4 h, 6 h, 8 h, 12 h, 16 h, and 24 h of imbibition. Subsequently, primed and over-primed seeds were collected at 24 h of hydropriming and during dry-back at 2 h, 4 h, and 6 h. (**b**) Percentage (%) of relative water content (RWC) during seed imbibition. (**c**) Percentage (%) of relative water content (RWC) during seed dry-back. Relative gene expression profiles of *MtSOG1a* (**d**) during seed imbibition and (**e**) priming (P)–over-priming (OP). Relative gene expression profiles of *MtSOG1b* (**f**) during seed imbibition and (**g**) priming (P)–over-priming (OP). Data were analyzed with two-way ANOVA and Tukey–Kramer test, where means without common letters are considered significantly different (*p* < 0.05).

For the seed germination system, primed and over-primed *M. truncatula* seeds, obtained through hydropriming followed by desiccation (dry-back), were analyzed at the indicated time points of imbibition and desiccation, respectively (Figure 6a). Relative water content (RWC) measurements indicate the levels of water uptake during imbibition (Figure 6b) and water loss during desiccation (Figure 6c). It is possible to observe that after dry-back, the RWC of primed seeds is similar to dry (unprimed) seeds while the over-primed seeds retain more water, and the desiccation curve is slowed down. Looking at the expression profiles of *MtSOG1a* during seed imbibition, it is possible to observe significant upregulation only from 12 to 24 h (Figure 6d). Seed priming, on the other hand, anticipated the *MtSOG1a* upregulation, observed already in the 2-6 h interval (Figure 6e). Differently, no significant changes compared to the control (0 h) were observed during over-priming (OP), although the gene is significantly more expressed relative to priming conditions (P) at 4 h and 6 h of dry-back. A similar pattern is also observed when considering the expression of *MtSOG1b* during seed imbibition (Figure 6f), whereas a trend of increased expression in response to over-priming was observed only at 4 h of dry-back (Figure 6g). Why is it important to study such a system? Even though enhanced germination speed and uniformity are viewed as added value in agricultural contexts [75,76], the empirical choice of priming protocols can be conducive to inadequate imbibition–dehydration timing patterns that may operate at the limits of seed desiccation tolerance [77]. Limiting the occurrence of over-priming is, therefore, crucial to optimizing seed-priming protocols. Moreover, in the context of priming protocols, the occurrence of over-priming can be envisioned as a model to identify possible hallmarks of desiccation stress, which can include reactive oxygen species (ROS), expression profiles of genes involved in DNA damage (e.g., *OGG1*, *TDP1*), the antioxidant (e.g., *APX*, *SOD*), and DDR (e.g., *MRE11/RAD50/NBS1*) pathways [73,74]. The upregulation of *SOG1* in the context of seed imbibition is in line with previous work ascertaining that the DDR pathway is highly active during this process to allow the repair of DNA damage (possibly induced by ROS accumulation during water uptake) before the start of cell division [63,78]. Similarly, priming treatments have been already correlated with anticipated germination as well as anticipated gene expression profiles, specifically related to DNA damage repair [54,79]. 

**Figure 7:**
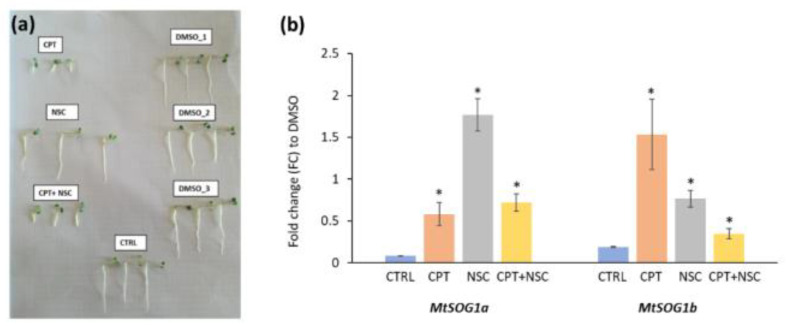
(**a**) Representative image of 7-day-old *M. truncatula* seedlings to evaluate the phenotypic effect of CPT, NSC, and CPT+NSC treatments and corresponding DMSO concentrations (DMSO_1, DMSO_2, DMSO_3). Control (CTRL), non-treated seedlings are also included. (**b**) Expression profiles of *MtSOG1a* and *MtSOG1b* in response to the imposed treatments. Data are represented as fold-change (FC) to the respective DMSO controls. Statistically significant (*p* < 0.05) differences between treatments and control (CTRL, un-treated seedlings) are represented with an asterisk (*).

The CPT/NSC system, applied to 7-day-old seedlings, grown in the presence of CPT, NSC, and CPT+NSC, and their corresponding DMSO controls, resulted in changes in seedling morphology for the CPT and CPT+NSC treatments, where seedlings were shorter and stockier compared to their respective controls (Figure 7a). To investigate the effect of CPT/NSC treatments, the relative expression values obtained for *MtSOG1* genes are presented as the fold-change (FC) to each correspondent DMSO concentration (Figure 7b). The results show that both *MtSOG1a* and *MtSOG1b* genes are upregulated in response to the imposed treatments. Nonetheless, the degree of upregulation is different between the two genes during the treatments. While the *MtSOG1a* gene expression is highly induced by the NSC treatments, the *MtSOG1b* gene expression is highly triggered by the CPT treatment. Differential expression of other genes involved in DNA damage repair (e.g., *PARP1*, *ERCC1*, *MUS81*, *MRE11/RAD50/NBS1*) and cell-cycle regulation (*Cdk1a*, *Cycb1*, *Cycd2*, *TOR*) were also observed in this system, suggesting that the imposed treatments can truly affect DDR [53]. Differently from the usual treatments known to induce DSBs and the activation of DDR (e.g., irradiation, zeomycin), the relevance of using such a system of treatments comes from the different types of DNA damage (e.g., DNA–protein crosslinks, oxidative damage) and respective DNA repair pathways that are activated. While CPT treatments are known to activate the DNA–protein crosslink repair (DPC) [80], NSC treatments were connected to the activation of Base Excision repair (BER) [81]. These effects may rely on the existing interactions between players such as TopI (topoisomerase 1, an enzyme that cut one of the two strands of double-stranded DNA, resulting in the accumulation of single-strand breaks) and TDP1 (tyrosyl DNA phosphodiesterase 1, involved in the removal of TopI-DNA covalent complexes). During the CPT treatment, *TDP1* and *TopI* genes are inhibited while genes involved in DNA repair pathways alternative to TDP1 (e.g., *PARP1*, *ERCC1*, *MUS81,*) are highly active. When NSC is given, the *TDP1* and *Top1* genes are active, and the alternative repair is inhibited. The NSC+CPT combination target both TDP1 and TopI functions, resulting in cytotoxic and genotoxic effects, corresponding to the obstructed seedling development [53]. 

Overall, the registered data aptly corroborate the data retrieved from the public repository as well as complement the recent study on *P. patens* [27], suggesting that the multiple *SOG1* homologues have redundant functions in a given plant species. 

Why the *SOG1* function has developed this redundancy in plant species is still a question to be answered in future works. Nonetheless, there are some indications related to other redundancies also related to the DDR pathway in the mammalian system. It is believed that, to avoid failure of its essential function in maintaining genome stability, different steps of DDR can be highly redundant and employ different mechanisms [82,83]. The fact that this redundancy is spread across all kingdoms may imply the high importance that is placed on the detection and repair of DNA damage during evolution [84,85]. Specifically related to plants, the fact that there are multiple SOG1 homologues with redundant functions could contribute to improving the ability of a given species to respond to environmental stresses. Alternatively, this may represent a buffering strategy, wherein in the case one gene becomes inactive, the others guarantee the function as evidenced for other gene families [86].

## 4. Conclusions

In the current work, multiple SOG1 genes were identified for the following plant species: *B. rapa*, *G. max*, *K. fedtschenkoi*, *K. laxiflora*, *L. usitatissimum*, *M. domestica*, *M. esculenta*, *M. truncatula*, *P. thricocarpa*, *P. virgatum*, *S. italica*, *S. purpurea*, and *S. viridis*, based on data gathered from Phytozome. The phylogenetic analyses revealed two main groups for Monocots and Eudicots species, along with the presence of the NAM (more recently referred to as NAC) domain and the specific SQ motives (required for ATM/ATR phosphorylation) in all analyzed species.

A gene expression data mining approach carried out in four specie (*M. truncatula*, *G. max*, *K. fedtschenkoi*, *P. trichocarpa*) possessing multiple *SOG1* homologues indicated that these have similar expression patterns under different conditions, suggesting redundant functions in plants as recently proposed in *P. patens* [27].

Data retrieved from in silico protein–protein interactors showed that predicted interactomes of SOG1 are overlapping (between homologues of the same species as well as between different species) and mostly include proteins with demonstrated functions in DDR and DDR-downstream pathways (e.g., DNA repair, cell-cycle checkpoint).

Finally, experimental systems designated to target seed germination and seedling responses to genotoxic stresses were applied to support the gene expression data-mining approach, using the model legume *M. truncatula*. This corroborates the redundant function of the two genes (*MtSOG1a* and *MtSOG1b*) which followed a comparable expression pattern, being upregulated during seed imbibition as well as CPT/NSC treatments. Moreover, the retrieved data on early seed development and generated data on early seed germination agree with the required activation of DDR during these physiological processes. 

## Data Availability

Not applicable.

## Supplementary Materials

The following supporting information can be downloaded at: https://www.mdpi.com/article/10.3390/genes13040667/s1, Figure S1: Sequence alignment between SOG1 amino acid sequences from *A. thaliana*, *M. truncatula*, *G. max*, *K. fedschenkoi*, *P. trichocarpa*; Table S1: Percentage (%) of similarity between SOG1 amino acid sequences from *A. thaliana*, *M. truncatula*, *G. max*, *K. fedschenkoi*, *P. trichocarpa*; Table S2: Lists of putative *cis*-acting regulatory elements identified in the promoter regions of *MtSOG1a* and *MtSOG1b*; Table S3: Lists of putative protein–protein interactors, identified via STRING, provided for *A. thaliana* AtSOG1, *M. truncatula* MtSOG1 and MtSOG1b, *G. max* GmSOG1a, GmSOG1b, GmSOG1c, GmSOG1d, and *P. trichocarpa* PtSOG1a and PtSOG1b.
