# Supplementary material for: Identification and Characterization of SOG1 (Suppressor of Gamma Response 1) Homologues in Plants Using Data Mining Resources and Gene Expression Profiling
Source: Genes (Basel). 2022 Apr 9;13(4):667. doi: 10.3390/genes13040667 (PMC9026448; doi:10.3390/genes13040667)
Supplement: Supplementary file 1 [file genes-13-00667-s001.zip › Pagano et al_Supplementary material.pdf]

# Supplementary Material

## Identification and Characterization of SOG1 (Suppressor of Gamma-Response 1) Homologues in Plants Using Data Mining Resources and Gene Expression Profiling

Andrea Pagano[1], Carla Gualtieri[1,#], Giacomo Mutti[1,§], Alessandro Raveane[1,‡], Federico Sincinelli[1], Ornella Semino[1], Alma Balestrazzi[1], Anca Macovei[1,*]

[1] Department of Biology and Biotechnology 'L. Spallanzani', University of Pavia, via Ferrata 9, 27100 Pavia, Italy

Present addresses:

[#] Department of Agricultural, Forest and Food Sciences, University of Turin, via Verdi 8, 10124 Turin, Italy

[§] Department of Chemistry, Life Sciences and Environmental Sustainability, University of Parma, Parco Area delle Scienze 11/a, 43124, Parma, Italy

[‡] Human Technopole, Viale Rita Levi-Montalcini 1, 20157 Milan, Italy

* Corresponding author: A. Macovei, e-mail: anca.macovei@unipv.it

```
A.thaliana_AT1G25580                --------------------MAGRSWLIDSNRIATKIMSASASSDPRQVVWKSNPSRHCP      40
M.truncatula_Medtr1g093680          ------------------MAASGPSWLVDKRRIATKIKRASEAT--EKVIWESNPTRACP      40
G.max_Glyma.10G204700               --------------------MAGPSWLVDKSRIATKIKNVSGTC--GKVIWKSNPSRACP      38
G.max_Glyma.20G185800               MLKTILNLSHLIASFSDLFPMAGPSWLVDKSRIATKIKSASGTC--GKVIWKSNPSRACP      58
M.truncatula_Medtr5g053430          --------------------MAGPSWLVDKSRIATKIKSASGACDPENVTWKSNPTKACP      40
G.max_Glyma.01G088200               --------------------MAGTSWLVDKSRIATKIKCASGACDPGKVIWKSNPTKPCP      40
G.max_Glyma.02G100200               --------------------MAGTSWLVDKSRIATKIKCASGACDHGKVIWKSNPTKACP      40
K.fedtschenkoi_Kaladp0073s0030      -------------------MSTGPTWLVDGHRIATKIKSAAGASDLGHMKWKSNPCRACP      41
K.fedtschenkoi_Kaladp0062s0046      --------------------MARPAWLVDSTRIATKIKSASTACPPESINWKSNPSRICP      40
P.trichocarpa_Potri.008G116600      ---------------------MAGPWLVDGNRFATKIKSASSASNHERVAWKSDPCKTCP      39
P.trichocarpa_Potri.010G129700      ---------------------MAGPWLVDGNRFATKIKSASGASNLERVEWKSDPCKTCP      39
                                                             **:*  *:****  .: :     : *:*:* : **

A.thaliana_AT1G25580                KCQHVIDNSDVVDDWPGLPRGVKFDPSDPEIIWHLLAKSGLSGLSSHPFIDEFIPTVNQD      100
M.truncatula_Medtr1g093680          NCQHVIDNSDVTQEWPGLPKGVKFDPSDQEIIWHLLAKVGEGNLEPHPFIDEFISTLQMD      100
G.max_Glyma.10G204700               SCHHVIDNSDVAQEWPGLPLGVKFDPSDQEIIWHLLAKVGAGNSKPHPFIDEFITSLEVD      98
G.max_Glyma.20G185800               SCHHVIDNSDVAQEWPGLPIGVKFDPSDQEIIWHLLAKVGAGNSKPHPFIDEFITTLEVD      118
M.truncatula_Medtr5g053430          NCQHTIDNSDVVKEWPGLPKGVKFDPSDQEVIWHLLAKCGVGNSKPHPFVDEFIITLEVN      100
G.max_Glyma.01G088200               NCQHAIDNDDVAQEWPGLPKGVKFDPSDQEIIWHLLAKVGVGDSKPHPFIDEFITTLEVD      100
G.max_Glyma.02G100200               NCQHAIDNGDVAQEWPGLPKGVKFDPSDQEIIWHLLAKVGVGDSKSHPFIDEFITTLEVD      100
K.fedtschenkoi_Kaladp0073s0030      NCQYIIDNSDVPQDWPGLPRGVKFDPTDQEIIHHLLAKCGIGNLKAHPFINEFIPTVNKE      101
K.fedtschenkoi_Kaladp0062s0046      NCQHCIDNSDVPQEWPGLPRGVKFDPSDQEIIWHLLTKVDSTNLTPHPFIDEFIATVDKD      100
P.trichocarpa_Potri.008G116600      NCHHVIDNSDVVQEWPGLPRGVKFDPSDQEIIWHLLAKAGNGGIKSHPFIKEFIPTVEND      99
P.trichocarpa_Potri.010G129700      NCHHVIDNSDVVQEWPGLPRGVKFDPSDQEIIWHLLAKVSDGGIKPHPFIKEFIPTVDND      99
                                    .*:: ***.** .:***** ******:* *:* ***:* .  .   ***:.*** ::: :

A.thaliana_AT1G25580                DGICYTHPKNLPGVKSDGTVSHFFHKAIKAYSTGTRKRRKIHDDDFGDVRWHKTGRTKPV      160
M.truncatula_Medtr1g093680          DGICYTHPKRLPGVSQDGIASHFFHRAIKAYNTGSRKRRKICGQDFGDVRWHKTGRTKPI      160
G.max_Glyma.10G204700               DGICYTHPQHLPGVKQDGSASHLFHRAIKAYNTGSRKRRKICGQDFGDVRWHKTGRTKPV      158
G.max_Glyma.20G185800               DGICYTHPQHLPGVKQDGSSSHFFHRVIKAYNTGSRKRRKICGQDFGDVRWHKTGRTKPV      178
M.truncatula_Medtr5g053430          DGICYTHPQNLPGVKQDGNASHFFHRAIKAYNTGTRKRRKIHDQDFGDVRWHKTGRTKPV      160
G.max_Glyma.01G088200               DGICYTHPQNLPGVKQDGSASHFFHRAINAYNTGTRKRRKILGQDFGDVRWHKTGRTKPV      160
G.max_Glyma.02G100200               DGICYTHPQNLPGVRQDGSASHFFHRAIKAYNTGTRKRRKILGQDFGDVRWHKTGRTKPV      160
K.fedtschenkoi_Kaladp0073s0030      DGICYTHPQNLPGVKLDGSVAHFFHRAIKAYNTGSRKRRKIQGDSSGDVRWHKTGRTKPV      161
K.fedtschenkoi_Kaladp0062s0046      DGICYTHPQNLPGVKQDGSVTHFFHRAIKAYNSGTRKRRKIHGDDASDVRWHKTGRTKPV      160
P.trichocarpa_Potri.008G116600      DGICYTHPKNLPGVKQDGSISHFFHRAIKAYNTGTRKRRKIQGDDSGDVRWHKTGRTKPV      159
P.trichocarpa_Potri.010G129700      DGICYTHPQNLPGVKQDGSISHFFHRAIKAYNTGTRKRRKIQGDNFGDVRWHKTGRTKPV      159
                                    ********:.****  **  :*:**:.*:**.:*:****** .:. .************:

A.thaliana_AT1G25580                VLDGVQRGCKKIMVLY-----GGKAVKTNWVMHQYHLGIEEDEKEGDYVVSKIFYQQPQQ      215
M.truncatula_Medtr1g093680          LLNGLQKGCKKIMVLYMTLESGGKAEKTNWVMHQYHLGRQEDEKDGEYVISKVFYQQQQ-      219
G.max_Glyma.10G204700               ILNGVQKGCKKIMVLYISPVRGGKPEKTNWVMHQYHLGTEEDEKDGEYVISKVFYQQQQ-      217
G.max_Glyma.20G185800               ILNGIQKGCKKIMVLYISPVRGGKSEKTNWVMHQYHLGTEEDEKDGEYVISKVFYQQQQ-      237
M.truncatula_Medtr5g053430          ILNGVQKGCKKIMVLYMSSAGGGKAEKTNWVMHQYHLGTEEDEKDGEFVISKVFYQQQQ-      219
G.max_Glyma.01G088200               VFNGIQKGCKKIMVLYVSNVRGGRAEKTNWVMHQYHLGTEEDEKDGEYIISKVFYQQQQ-      219
G.max_Glyma.02G100200               VLSGVQKGCKKIMVLYVSNVRGGKAEKTNWVMHQYHLGTEEDEKDGEYIISKVFYQQQQ-      219
K.fedtschenkoi_Kaladp0073s0030      MLDGVQKGCKKIMVLYISPAKGGKAEKTNWVMHQYHLGTEEDEKDGECVISKIFYQQQP-      220
K.fedtschenkoi_Kaladp0062s0046      MLDGVQRGCKKIMVLYMTAAKGGKAEKTNWVMHQYHLGTEEDEKDGEYVISKVFYQQNQ-      219
P.trichocarpa_Potri.008G116600      ILDGVQKGCKKIMVLYTSTVRGGKAEKTNWVMHQYHLGTGEDERDGEYVVSKVFYQQQA-      218
P.trichocarpa_Potri.010G129700      ILDGIQKGCKKIMVLYMSTVRGGKAEKTNWVMHQYHLGTSEDERDGEYVVSKIFYQQQ--      217
                                    ::.*:*:*********     **:  ************  ***::*: ::**:****

A.thaliana_AT1G25580                LVVKRGDKAEQEVSEDIF------------AAVTPTADPVTPKLATPEPRNAVRICSDSH      263
M.truncatula_Medtr1g093680          --AKLGEKDDQNVPETT-------------EATVVKVDPVTPNSVTPEPPRNEKHCSDVD      264
G.max_Glyma.10G204700               --VKFGEKGDQDIPETT-------------EATTVKVDPVTPKSVTPEPPRIENRWSNLD      262
G.max_Glyma.20G185800               --VKFGEKDDQDIPEIT-------------EATTVKVDPATPKSVTPEPPRIEKRCSNLD      282
M.truncatula_Medtr5g053430          --VKLGDKDEQDIPEAI-------------EATIAKVDPVTPKSVTPEPPYTERLYSDVD      264
G.max_Glyma.01G088200               --VKLGDKDDKDVPEATEIN----------ESTIVKVDPVTPKFVTPETPCNERWCADLD      267
G.max_Glyma.02G100200               --VKLGDKNDQDVPEASEITGPHPGLSVGEEGTIEKVDPVTPKLVTPEPPCNGRWCADLD      277
K.fedtschenkoi_Kaladp0073s0030      --GKASDGVEQEISETS-------------NTAVAKVDPVTPRSAIPVPPRTEARDSDLE      265
K.fedtschenkoi_Kaladp0062s0046      --AKLGENFEAELMEIS-------------DLAAQKVDPLTPKSVSPEPRRDERPYAELG      264
P.trichocarpa_Potri.008G116600      --ANKGEKTEEDLSEIA-------------NAVVAKVDPVTPKSVTPDPPRAERRCQDFD      263
P.trichocarpa_Potri.010G129700      --ANKGEKPEEDLSEIV-------------DDVVAKVDPVTPKSVTPDPPRAERRCQDFE      262
                                       : .:  : :: *                 .  ..** **. . *          :

A.thaliana_AT1G25580                IASDYVTPSDYVSAHEVSLAETSEVMCMEDEVQSIQP-----------NHERPSSGPELE      312
M.truncatula_Medtr1g093680          QEQESHHSPQ------I-LPNLSRLEWLLESQDDCEELAKTDSSMIETQTNVGVDNFDLS      317
G.max_Glyma.10G204700               LGQESHHIPQ------H-----PQTDCLEDLQADCEDLAKDDLLIVETENNEGI------      305
G.max_Glyma.20G185800               LGQESHHIPQ------H-----PQTDYLDDLQAVCEDLAKDDLLIVETQNNEAI------      325
M.truncatula_Medtr5g053430          LGQDTPAIPQ------P-----PQMNCLDEIQPYCEEQAKDDLSMVETERNEGKDNNENN      313
G.max_Glyma.01G088200               LGQETHNSPQ------L-----PQMDCLDEIQADYQDLAN-ESSMVETQHNEGM------      309
G.max_Glyma.02G100200               LGQETHNVPQ------P-----PRMDCLDEIQADCQVLAN-ESSMVETQHNEGM------      319
K.fedtschenkoi_Kaladp0073s0030      LVEESALTQM------EPLHQGLDTD-YTGDLDEQN--ESEAIKIQDQPRAEG-------      309
K.fedtschenkoi_Kaladp0062s0046      NIDEATDVNV------ESFAQDQLEADYVTNADL-E--ETMDLRNQQLLNE---------      306
P.trichocarpa_Potri.008G116600      LGKESTNVCT------EPYAEHPGVENLEE-ANL-Q--HE-SPCYNDQPGM---------      303
P.trichocarpa_Potri.010G129700      MGQESTNICT------DPCAQQPGIECLEEEVNL-L--QK-NLSYVDQLET---------      303
                                      .:

A.thaliana_AT1G25580                HGLENGAK-EMLDD--KEEQEKDRDNENQGEEDPTWFDSGSQFILNSQQLVEALSLCDDL      369
M.truncatula_Medtr1g093680          HNLLDSQQLESQDDWEELAKTDSSTVETQTNVGVDNF-DLSQNLLDSQQLAEGLSLFADF      376
G.max_Glyma.10G204700               -------------------DN----IENNAEEDSKWWDSESQNLLSSQQLVEALFLCDDL      342
G.max_Glyma.20G185800               -------------------DN----IENNAEEDSKWWDSESQNLLSSQQLVEALFLCDDL      362
M.truncatula_Medtr5g053430          AD--------DGQKWCEGTDN----NENNADEGQKWWDSESQNLLDSQQLVDALSLCDDL      361
G.max_Glyma.01G088200               -------------------DD----KEKNTEEGQKWWDSESQNLLDSQQLVEALSLCDDL      346
G.max_Glyma.02G100200               -------------------DD----KENNAEDGQKWWDSESQNLLDSQQLVEALSLCDDL      356
K.fedtschenkoi_Kaladp0073s0030      ---------------HSGEGDAVENNDNPAAEDPSWWE-ESPFLLNSQQLAEGLAICDDL      353
K.fedtschenkoi_Kaladp0062s0046      ---------------ENALDDLMDYNDINAGEGQKWWDSESQNLLDSQQLVEGLSLCDDL      351
P.trichocarpa_Potri.008G116600      ---------------ENHVDQMVGDNDNCAEEGTKWWDSESQHLLDSQQLVEGLSLCDEF      348
P.trichocarpa_Potri.010G129700      ---------------ENNVNHMVGDNDNCAGEERKWWDSESQQLLDSQQLVEGLSLCAEF      348
                                                              :         :   *  :*.****.:.* :  ::

A.thaliana_AT1G25580                LGSQDREENTNSG--SLKDKQPCIADYAHLGPEDFKRDLEECQKIVLDPSNIELD-----      422
M.truncatula_Medtr1g093680          FQSQSPKRDGESG---EQMERPSLSVYAHLGAEQLKKDIEECQNLELNTTKKNDIEECLN      433
```

```
G.max_Glyma.10G204700               LQSQSPKRDGEN----EHKKRSSLSVYANLGPEHLKKDIVECQNLVLDTANV--------     390
G.max_Glyma.20G185800               LQSQSPKRDEEN----EHKKRPSLSVYADLGPEHLKKDIEECQNLVLDPANV--------     410
M.truncatula_Medtr5g053430          LRSQSPTRDGKDE---EQKNQPSLSIYAQLGPEHLKKDIEDCQNLVLEPSNVES------     412
G.max_Glyma.01G088200               LQSQSPSRDGKHE---DHKNQPGLSVYAQLGPENLKKDIEECQNLALDKAN---------     394
G.max_Glyma.02G100200               LQSQSPSRDGKHE---DHKNQPGLSVYAQLGPEHLKKDIEECQNLALNPAN---------     404
K.fedtschenkoi_Kaladp0073s0030      LQSQSPGRQGFA-NPQESKGRPRLADYSRLGPENLKKDLEECQTLEFDPTKIELD-ALLE     411
K.fedtschenkoi_Kaladp0062s0046      LRSQSPSRDFAVPEPHKPKAKPCLSDYATLGPNDLKRDLEECQKLVLDPANIEID-T---     407
P.trichocarpa_Potri.008G116600      LQSQSPSRGGHGT---GASGKSGLSDYARLGPEHLKKDLEECQNLELDPANIELD-T---     401
P.trichocarpa_Potri.010G129700      LQSQSPDRGGHGT---EANGKTGLSDYALLGPEYLKKDLEECQNLELDQANIELD-T---     401
                                    : **.  .            :  :: *: ** : :*:*: :**.: :: ::

A.thaliana_AT1G25580                --------------------TPPEFRLSQLEFGSQDSFL---------------AWGTGK     447
M.truncatula_Medtr1g093680          LDVETEKNLNLDTANVELDSQPSDIRLSQLEFSSQDSYI---------------AWGGCK     478
G.max_Glyma.10G204700               ----------------VPETPPSEFQLSQLDFSSQDSFV---------------SWCASK     419
G.max_Glyma.20G185800               ----------------VPETPPSEFRLSQLDFSSQDSFV---------------SWCANK     439
M.truncatula_Medtr5g053430          ---------------NVENDTPSEFRLSQLEFGSQDSFI---------------AFGYNK     442
G.max_Glyma.01G088200               ----------------IENDTPSEFRLSQLFSCFIGIWFTGQLYFLGLWQGSELIWFFTS     438
G.max_Glyma.02G100200               ----------------IENDTPSEFQLSQLEFGSQDS---------------FISWGYSK     433
K.fedtschenkoi_Kaladp0073s0030      LH------------LSQDIRLSQDFRLSQLEFCSQDSFC---------------AFGGNK     444
K.fedtschenkoi_Kaladp0062s0046      ---------------------PPDYRLSQLEFGSQDSFL---------------AWGGHK     431
P.trichocarpa_Potri.008G116600      ---------------------PPEFRLSQLEFGSQDSFM---------------AWGEGR     425
P.trichocarpa_Potri.010G129700      ---------------------PPEFRLSQLVCFLPHCFL---------------LSSV*-     423
                                                       : :****

A.thaliana_AT1G25580                TD*----------- 449
M.truncatula_Medtr1g093680          TIN*---------- 481
G.max_Glyma.10G204700               AFD*---------- 422
G.max_Glyma.20G185800               AID*---------- 442
M.truncatula_Medtr5g053430          VVK*---------- 445
G.max_Glyma.01G088200               AGQADVFVTTFCC* 451
G.max_Glyma.02G100200               AVN*---------- 436
K.fedtschenkoi_Kaladp0073s0030      VID*---------- 447
K.fedtschenkoi_Kaladp0062s0046      LGD*---------- 434
P.trichocarpa_Potri.008G116600      VAD*---------- 428
P.trichocarpa_Potri.010G129700      -------------- 423
```

**Figure S1**. Sequence alignment between SOG1 amino acid sequences from *A. thaliana*, *M. truncatula*, *G. max*, *K. fedschenkoi*, *P. trichocarpa*, performed using CLUSTALW online tool.

**Table S1**. Percentage (%) of similarity between SOG1 amino acid sequences from *A. thaliana*, *M. truncatula*, *G. max*, *K. fedschenkoi*, *P. trichocarpa*. In red are evidenced % similarities among the target species and *A. thaliana* (AT1G25580). Letter in brackets (a,b,c,d) refer to the nomenclature given to SOG1 isoforms based on sequence similarity to *A. thaliana*.

| | | 1 (%) | 2 (%) | 3 (%) | 4 (%) | 5 (%) | 6 (%) | 7 (%) | 8 (%) | 9 (%) | 10 (%) | 11 (%) |
|---|---|---|---|---|---|---|---|---|---|---|---|---|
| 1 | AT1G25580 | 100 | 53.46 | 58.85 | 59.85 | 60.10 | 57.43 | 61.44 | 57.11 | 60.87 | 62.04 | 58.48 |
| 2 | Medtr1g093680 **(b)** | 53.46 | 100 | 69.36 | 69.74 | 65.84 | 66.90 | 69.38 | 55.23 | 58.08 | 59.20 | 58.23 |
| 3 | Glyma.10G204700 **(c)** | 58.85 | 69.36 | 100 | 93.60 | 75.36 | 74.76 | 77.51 | 60.91 | 63.26 | 66.18 | 65.01 |
| 4 | Glyma.20G185800 **(b)** | 59.85 | 69.74 | 93.60 | 100 | 76.07 | 75.71 | 77.27 | 61.72 | 64.96 | 66.91 | 66.00 |
| 5 | Medtr5g053430 **(a)** | 60.10 | 65.84 | 75.36 | 76.07 | 100 | 76.36 | 80.52 | 60.80 | 65.00 | 65.71 | 64.08 |
| 6 | Glyma.01G088200 **(d)** | 57.43 | 66.90 | 74.76 | 75.71 | 76.36 | 100 | 88.97 | 56.12 | 61.89 | 64.39 | 64.44 |
| 7 | Glyma.02G100200 **(a)** | 61.44 | 69.38 | 77.51 | 77.27 | 80.52 | 88.97 | 100 | 59.52 | 64.88 | 68.63 | 64.76 |
| 8 | Kaladp0073s0030 **(b)** | 57.11 | 55.23 | 60.91 | 61.72 | 60.80 | 56.12 | 59.52 | 100 | 61.25 | 63.29 | 60.95 |
| 9 | Kaladp0062s0046 **(a)** | 60.87 | 58.08 | 63.26 | 64.96 | 65.00 | 61.89 | 64.88 | 61.25 | 100 | 64.72 | 62.88 |
| 10 | Potri.008G116600 **(a)** | 62.04 | 59.20 | 66.18 | 66.91 | 65.71 | 64.39 | 68.63 | 63.29 | 64.72 | 100 | 84.12 |
| 11 | Potri.010G129700 **(b)** | 58.48 | 58.23 | 65.01 | 66.00 | 64.08 | 64.44 | 64.76 | 60.95 | 62.88 | 84.12 | 100 |

**Table S2***. Lists of putative *cis*-acting regulatory elements identified in the promoter regions of *MtSOG1a* and *MtSOG1b* obtained using New PLACE Database (https://www.dna.affrc.go.jp/PLACE/?action=newplace). The site name, the position within the query sequence, the DNA strand (+ or -) within the query sequence, the consensus signal sequence and the site code are provided. A comparison between the putative *cis*-acting regulatory elements identified in the promoter regions is also given. The site name, the consensus signal sequence, the site code, and the number of copies identified in both promoters are provided along with annotations retrieved from New PLACE.

**Table S3***. Lists of putative protein-protein interactors, identified via STRING ((https://string-db.org/), provided for *A. thaliana* AtSOG1, *M. truncatula* MtSOG1 and MtSOG1b, *G. max* GmSOG1a, GmSOG1b, GmSOG1c, GmSOG1d, and *P. trichocarpa* PtSOG1a and PtSOG1b. A comparative analyses between the predicted interactors af all the proteins from the different species is also provided.

* Tables S2 and S3 are provided as separated excel files with multiple sheets.
